# Evaluating the role of land cover and climate uncertainties in computing gross primary production in Hawaiian Island ecosystems

**DOI:** 10.1371/journal.pone.0184466

**Published:** 2017-09-08

**Authors:** Heather L. Kimball, Paul C. Selmants, Alvaro Moreno, Steve W. Running, Christian P. Giardina

**Affiliations:** 1 College of Arts and Sciences, University of Hawaii at Hilo, Hilo, Hawaii, United States of America; 2 U.S. Geological Survey, Western Geographic Science Center, Menlo Park, California, United States of America; 3 University of Montana, Numerical Terradynamic Simulation Group, Missoula, Montana, United States of America; 4 USDA Forest Service, Institute of Pacific Islands Forestry, Hilo, Hawaii, United States of America; Montana State University Bozeman, UNITED STATES

## Abstract

Gross primary production (GPP) is the Earth’s largest carbon flux into the terrestrial biosphere and plays a critical role in regulating atmospheric chemistry and global climate. The Moderate Resolution Imaging Spectrometer (MODIS)-MOD17 data product is a widely used remote sensing-based model that provides global estimates of spatiotemporal trends in GPP. When the MOD17 algorithm is applied to regional scale heterogeneous landscapes, input data from coarse resolution land cover and climate products may increase uncertainty in GPP estimates, especially in high productivity tropical ecosystems. We examined the influence of using locally specific land cover and high-resolution local climate input data on MOD17 estimates of GPP for the State of Hawaii, a heterogeneous and discontinuous tropical landscape. Replacing the global land cover data input product (MOD12Q1) with Hawaii-specific land cover data reduced statewide GPP estimates by ~8%, primarily because the Hawaii-specific land cover map had less vegetated land area compared to the global land cover product. Replacing coarse resolution GMAO climate data with Hawaii-specific high-resolution climate data also reduced statewide GPP estimates by ~8% because of the higher spatial variability of photosynthetically active radiation (PAR) in the Hawaii-specific climate data. The combined use of both Hawaii-specific land cover and high-resolution Hawaii climate data inputs reduced statewide GPP by ~16%, suggesting equal and independent influence on MOD17 GPP estimates. Our sensitivity analyses within a heterogeneous tropical landscape suggest that refined global land cover and climate data sets may contribute to an enhanced MOD17 product at a variety of spatial scales.

## Introduction

Gross primary production (GPP), the rate at which atmospheric carbon dioxide is fixed by photosynthesis, is the largest carbon flux from the atmosphere to the terrestrial biosphere [1, 2]. Spatiotemporal variation in GPP directly influences atmospheric CO_2_ concentrations and the global climate, making continuous monitoring of GPP an essential component of international policy aimed at climate change mitigation [3–6]. A photosynthesis algorithm (PSN) incorporating satellite observations from the Moderate Resolution Imaging Spectrometer (MODIS) provided the first remote-sensing based global GPP data product at 1-km resolution (MOD17) [7]. The MOD17 data product is a widely-used tool for monitoring spatiotemporal trends in global GPP, but uncertainty likely increases at the regional scale due to reliance on coarse resolution climate and land cover data inputs to the PSN algorithm [8–10]. Understanding the uncertainty in remote-sensing based GPP estimates is important to consider when applying these estimates at different spatial scales [11–13]. Yet quantifying the uncertainty in MOD17 GPP estimates remains challenging because the uncertainties in land cover and climate input data are not themselves well quantified [14]. Here we examine how local, high-resolution land cover and climate data products influence MOD17 GPP estimates in the Hawaiian Islands, a tropical landscape that is spatially heterogeneous in both land cover and climate [15].

The MOD17 PSN algorithm estimates GPP using remotely sensed surface reflectance combined with a global land cover data product (MCD12Q1) [16,17] and coarse-resolution climate data products from the NASA Global Monitoring and Assimilation Office (GMAO) [18]. Land cover data is used to apply biome-specific radiation use efficiency (RUE) terms to convert absorbed photosynthetically active radiation (PAR) to GPP. Climate data is used to estimate incident PAR and to attenuate RUE based on temperature and moisture limitations. Therefore, any uncertainty in the land cover and climate data input products will propagate through to final MOD17 GPP estimates [10,14,19–21]. Globally, the accuracy of the MCD12Q1 land cover product is 65–80%, with higher overall accuracy in more homogeneous landscapes [17]. Similarly, the coarse resolution GMAO climate input data products (~0.5 degree) are recognized as major sources of uncertainty in the MOD17 GPP product [14] and likely reduce the accuracy of GPP estimates in areas such as the Hawaiian Islands with steep climatic gradients over short distances.

We evaluated the sensitivity of MOD17 GPP estimates to upstream data input from high-resolution Hawaii-specific land cover and climate products. Specifically, we compared MOD17 GPP estimates for the State of Hawaii based on four different combinations of land cover and climate input data: 1) global land cover and climate data from the currently available MOD17 product for Hawaii; 2) global land cover data paired with high-resolution Hawaii-specific climate data; 3) a Hawaii-specific land cover data product paired with global climate data; and 4) the combination of Hawaii-specific land cover and Hawaii-specific high-resolution climate data products. The lack of published independent ground-level GPP measurements in Hawaiian ecosystems means we cannot address whether employing local, high-resolution land cover and climate data inputs improves the accuracy of MOD17 GPP estimates in Hawaii. Nevertheless, our quantitative analysis of the errors associated with the coarse resolution global land cover and climate input data to the MOD17 algorithm provides insight into two sources of uncertainty for regional GPP estimates of heterogeneous landscapes.

## Methods

### Study area

We assessed the sensitivity of MOD17 GPP estimates to upstream land cover and climate data inputs by comparing GPP estimates based on global coarse resolution land cover and climate input data to GPP estimates based on Hawaii-specific, high-resolution land cover and climate input data. We conducted these analyses for the seven main Hawaiian Islands, listed here in descending order by percentage of total land area: Hawaii Island (63%), Maui (11%), Oahu (9%), Kauai (9%), Molokai (4%), Lanai (2%) and Kahoolawe (1%). The Hawaiian Islands are characterized by steep topographic relief, leading to large changes in climate over short distances. The eastern sides of islands receive abundant rainfall (2000–10,000 mm y^-1^) caused by exposure to moisture-laden trade winds and an inversion layer that caps orographic uplift, while the western, leeward sides of islands tend to be much drier (200–2000 mm y^-1^) [22]. The smaller islands of Kahoolawe and Lanai are located in the leeward rain shadow of Maui and tend to be drier. Mean annual temperatures range from 24°C at sea level to 4°C at 4200 m, yet seasonal variation in temperature at all points along this elevation gradient is minimal (± 1.5°C) [23]. The combination of steep climatic gradients and complex historical patterns of human land use and plant invasions have led to highly heterogeneous land cover within a very small geographic area [15].

### GPP models

The MOD17 PSN algorithm [7] for estimating GPP is based on the fraction of absorbed photosynthetically active radiation (fAPAR) calculated using the remotely-sensed fraction of incident photosynthetically active radiation (400–700 nm) absorbed by the canopy, which is collected by MODIS onboard the NASA Earth Observing System Aqua and Terra satellites [24,25]. Here we used version 6 of the fAPAR product calculated globally at a 500-m spatial resolution over an 8-day time step [7], along with a local linear regression gap filling model to reduce cloud contamination [26]. To calculate absorbed photosynthetically active radiation (APAR), the MOD17 PSN algorithm multiplies fAPAR by estimates of incident photosynthetically active radiation (PAR) from NASA GMAO (version 5.9.1) [27] produced at a spatial resolution of 0.5 Latitude degree by 0.67 Longitude degree. The MOD17 PSN algorithm resolves this inconsistency in spatial resolution between GMAO PAR and MODIS fAPAR input data by applying computationally efficient non-linear smoothing of the coarse resolution GMAO meteorological data to the 500-m MODIS pixel resolution [7]. To calculate GPP, APAR is multiplied by radiation use efficiency (RUE) terms specific to land cover classes designated in the global land cover product, MCD12Q1 [16,17,28,29]. These land cover specific RUE terms are attenuated during periods of low temperature or high vapor pressure deficit (VPD) by applying attenuation scalars that are simple linear ramp functions of daily minimum temperature and VPD (S1 Table) [7]. We compared GPP estimates from four models: 1) a global land cover and global climate model (GLGC) using the standard version 6 MOD17 product with global land cover (MCD12Q1) and global climate (GMAO) data products; 2) a global land cover and Hawaii climate model (GLHC); 3) a Hawaii land cover and global climate model (HLGC); and 4) a Hawaii land cover and Hawaii climate model (HLHC). All four GPP models were transformed to the WGS-84 geographic coordinate system using a custom Interactive Data Language (IDL) script (Harris Geospatial, Broomfield, CO).

The HLGC GPP model incorporates a high-resolution, Hawaii-specific land cover data product but uses the global GMAO product for PAR, VPD and temperature. The Hawaii land cover product, produced as part of the USGS Carbon Assessment of Hawaii [30], was developed at 30-m resolution incorporating HI-GAP [31], NOAA C-CAP [32], and LANDFIRE [33,34] land cover data products that were edited and validated using high-resolution imagery from World-View 2 [35] and Pictometry Online [36]. The Hawaii-specific land cover product identifies 48 land cover classes for Hawaii. We converted these land cover classes to the more general MCD12Q1 classes (S2 Table) and then performed a majority resampling of the Hawaii land cover product using ArcGIS v. 10.2.2 (ESRI, Redlands, CA) to produce a 500-m resolution product.

The GLHC model uses the global MCD12Q1 land cover data product but includes Hawaii-specific climate data products to compare the influence of global vs. local climate products on MOD17 estimates of GPP. Specifically, we incorporated Hawaii-specific estimates of incident PAR, mean annual temperature (MAT) and mean annual VPD from the Climate Atlas of Hawaii, a spatial interpolation of in situ meteorological measurements from over 1,000 climate stations in the State of Hawaii [23]. Data products from the Climate Atlas of Hawaii were resampled from 250-m to 500-m resolution. The GLGC model used the standard version 6 MOD17 product with global land cover (MCD12Q1) and global climate (GMAO) products, and the HLHC model incorporated both the Hawaii-specific land cover and Hawaii-specific climate data products as described above.

### Data analysis

To assess agreement between the Hawaii land cover data product and MCD12Q1, we first extracted pixel values from the two land cover products to a statewide 500-m point grid created in ArcGIS 10.2.2, resulting in approximately 70,000 records for the entire study area. We then calculated percent agreement and kappa coefficient [37] between the Hawaii land cover product and the MCD12Q1 land cover product using a confusion matrix generated from this point vector file (S3 Table). To compare the global and Hawaii-specific climate data products, we calculated summary statistics including the mean, standard deviation, range, and maximum and minimum values for PAR, VPD and MAT from both climate datasets. GPP values from each of the four GPP models were extracted to the 500-m point grid and compared by land cover class, by island, and statewide. We used one sample t-tests (α = 0.05) to evaluate whether GPP estimates from the GLGC model were significantly different from each of the three models incorporating Hawaii-specific data products (HLGC, GLHC, and HLHC) and we used Cohen’s D to calculate the individual effect sizes of using Hawaii-specific land cover and climate products on estimates of GPP, as well as the effect size of using the combination of both Hawaii-specific land cover and climate products on estimates of GPP. All statistical tests were conducted in R version 3.1.2 (2014).

## Results

**Fig 1:**
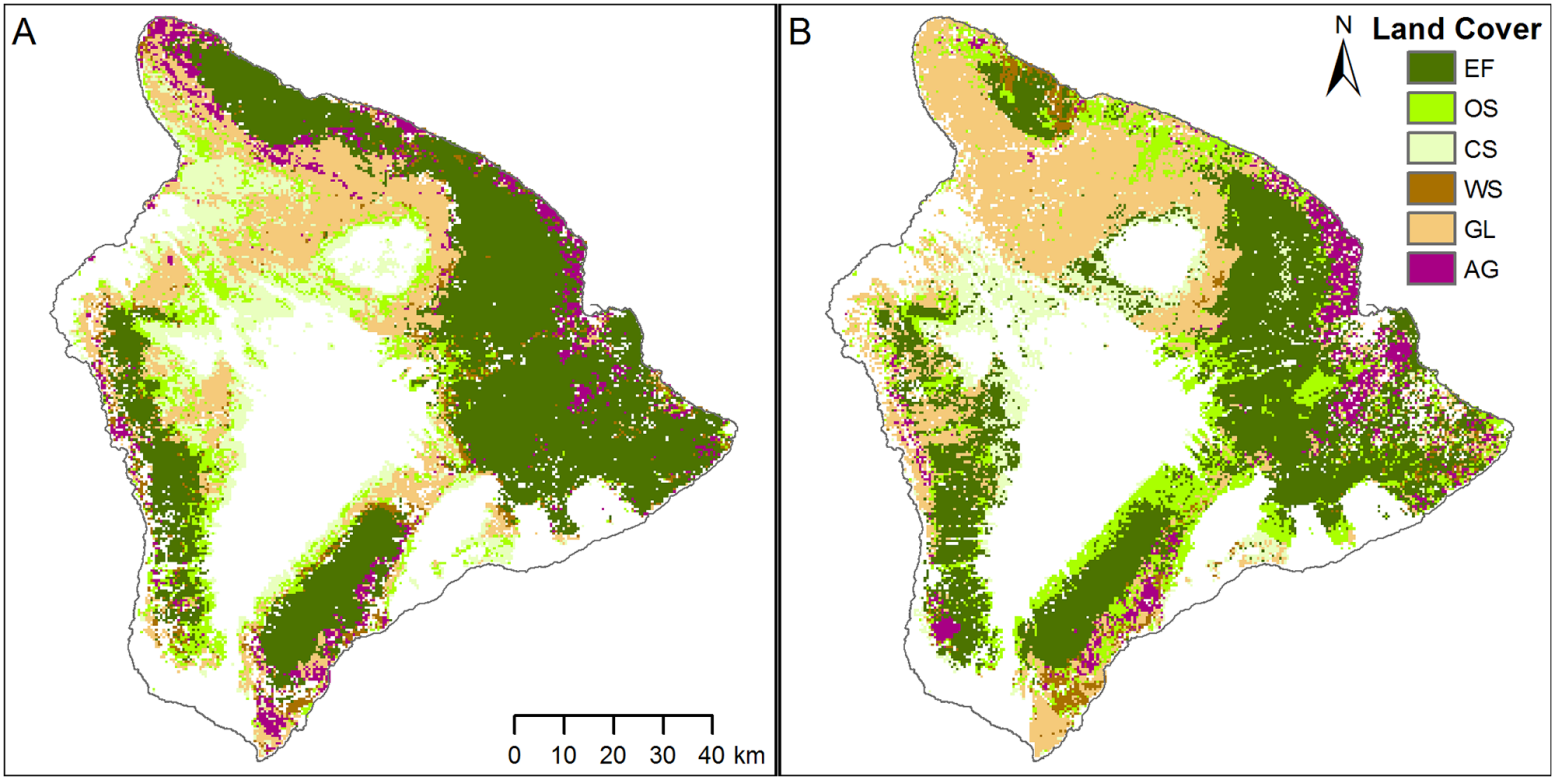
The global and Hawaii-specific land cover data products. (A) MCD12Q1 and (B) the Hawaii-specific land cover data product shown for Hawaii Island, the largest of the seven main islands of Hawaii. Land cover classes include evergreen broadleaf forest (EF), open shrubland (OS), closed shrubland (CS), woody savanna (WS), grassland (GL) and agriculture (AG). Remaining areas are sparse, barren or developed and are not used to estimate GPP.

**Fig 2:**
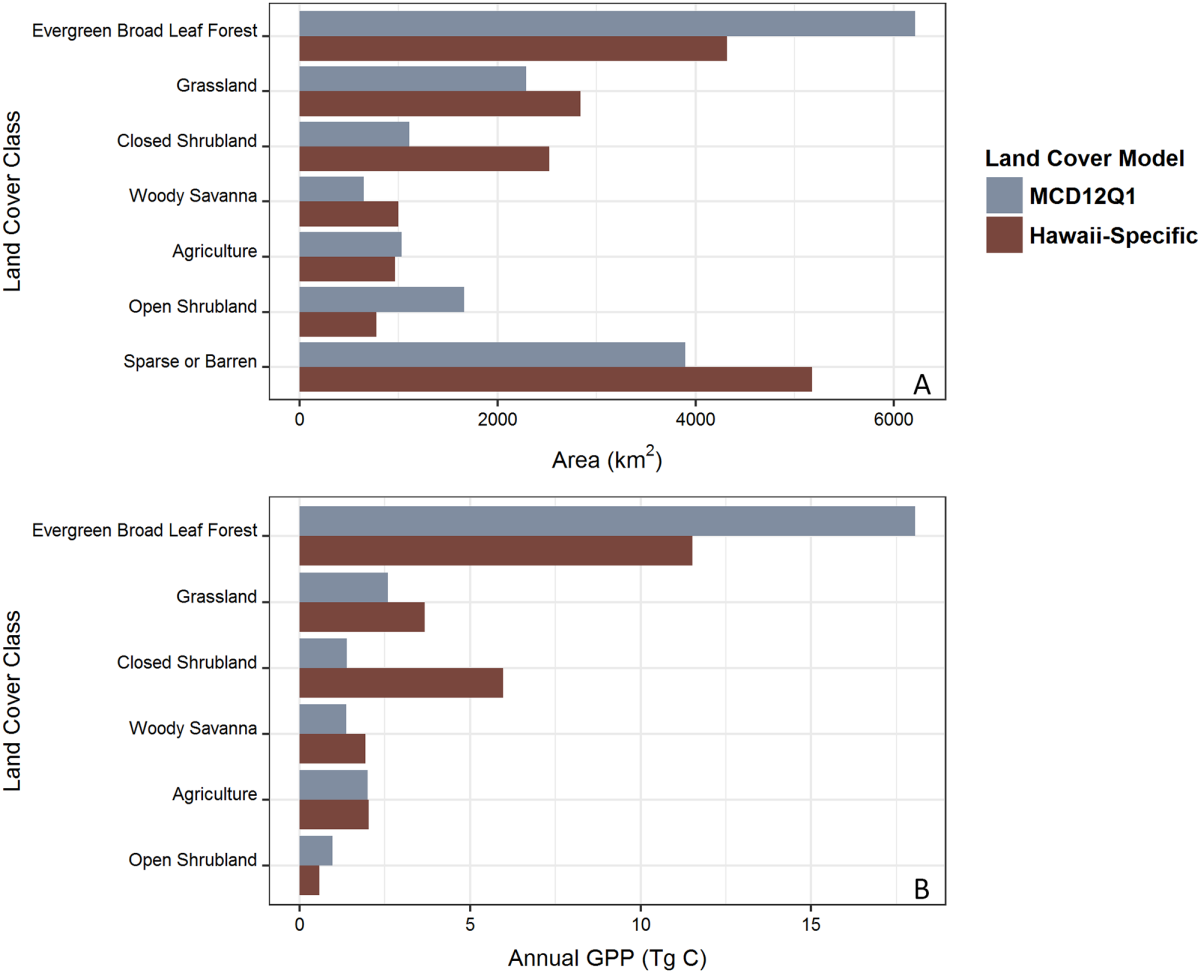
The area and contribution to statewide GPP of each land cover class by data product. (A) The area of each land cover class in the Hawaii-specific land cover data product and in MCD12Q1 for the main Hawaiian Islands. (B) The annual GPP for each land cover class in the GLGC model that used the global land cover data product, MCD12Q1 vs. the HLGC model, which uses the Hawaii-specific land cover data product. GPP values were not calculated for pixels classified as sparse or barren.

The overall percent agreement between the Hawaii land cover data product and MCD12Q1 was 51.6%, kappa = 0.44 (Fig 1; S3 Table). The evergreen broad leaf forest land cover class had the highest percentage agreement (80%) between the Hawaii and global land cover products (Table 1), yet there were one-third fewer pixels classified as evergreen broad leaf forest in the Hawaii land cover product than in MCD12Q1 (Fig 2A). Closed shrubland and woody savanna had the fewest pixels in agreement between the two land cover products (10%; Table 1). The sparse or barren classification in the Hawaii land cover product had a 68% agreement with MCD12Q1 (Table 1) but the Hawaii-specific land cover product included 25% more pixels in this classification than the MCD12Q1 product (Fig 2A).

**Table 1:** Alignment matrix between the Hawaii-specific and MCD12Q1 land cover products.

	MCD12Q1 Land Cover Designation						
Hawaii-specific Land Cover Designation	Evergreen Broad Leaf Forest	Closed Shrubland	Open Shrubland	Woody Savanna	Grassland	Agriculture	Sparse or Barren
Evergreen Broad Leaf Forest	**80**	5	3	2	4	1	1
Closed Shrubland	46	**10**	12	6	12	5	2
Open Shrubland	15	21	**43**	1	16	0	2
Woody Savanna	27	11	15	**10**	21	5	0
Grassland	19	7	15	4	**40**	9	0
Agriculture	34	3	1	6	12	**33**	0
Sparse or Barren	6	3	6	2	4	4	**68**

The percentage of pixels for each land cover class as classified in the Hawaii-specific land cover product that were classified in each corresponding class of the MCD12Q1 product. The values highlighted in grey represent the percentage of pixels in agreement between models for each class. Row values do not sum to 100% because some MCD12Q1 land cover classes were not represented in the Hawaii-specific land cover data product.

**Fig 3:**
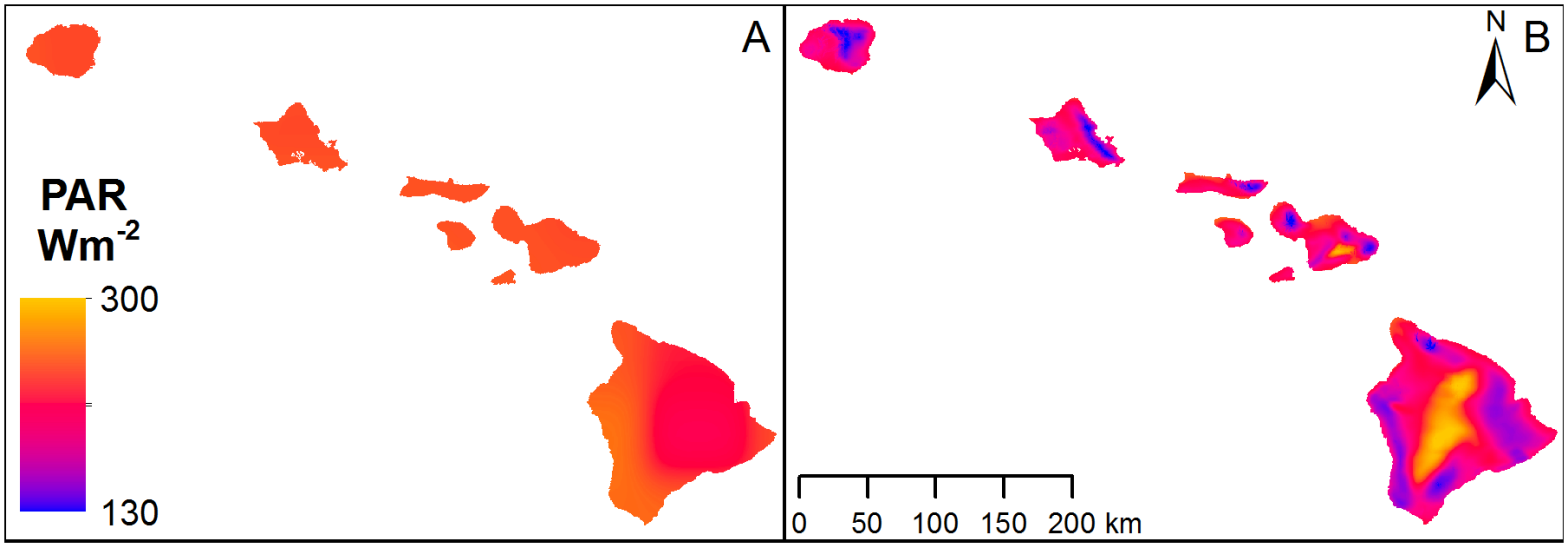
GMAO and Hawaii-specific data products for photosynthetically active radiation (PAR). (A) GMAO PAR used in the GLGC and HLGC GPP models and (B) Hawaii-specific PAR from the Climate Atlas of Hawaii used in GLHC and HLHC GPP models, both shown at the 130–300 W m^-2^ range scale.

**Fig 4:**
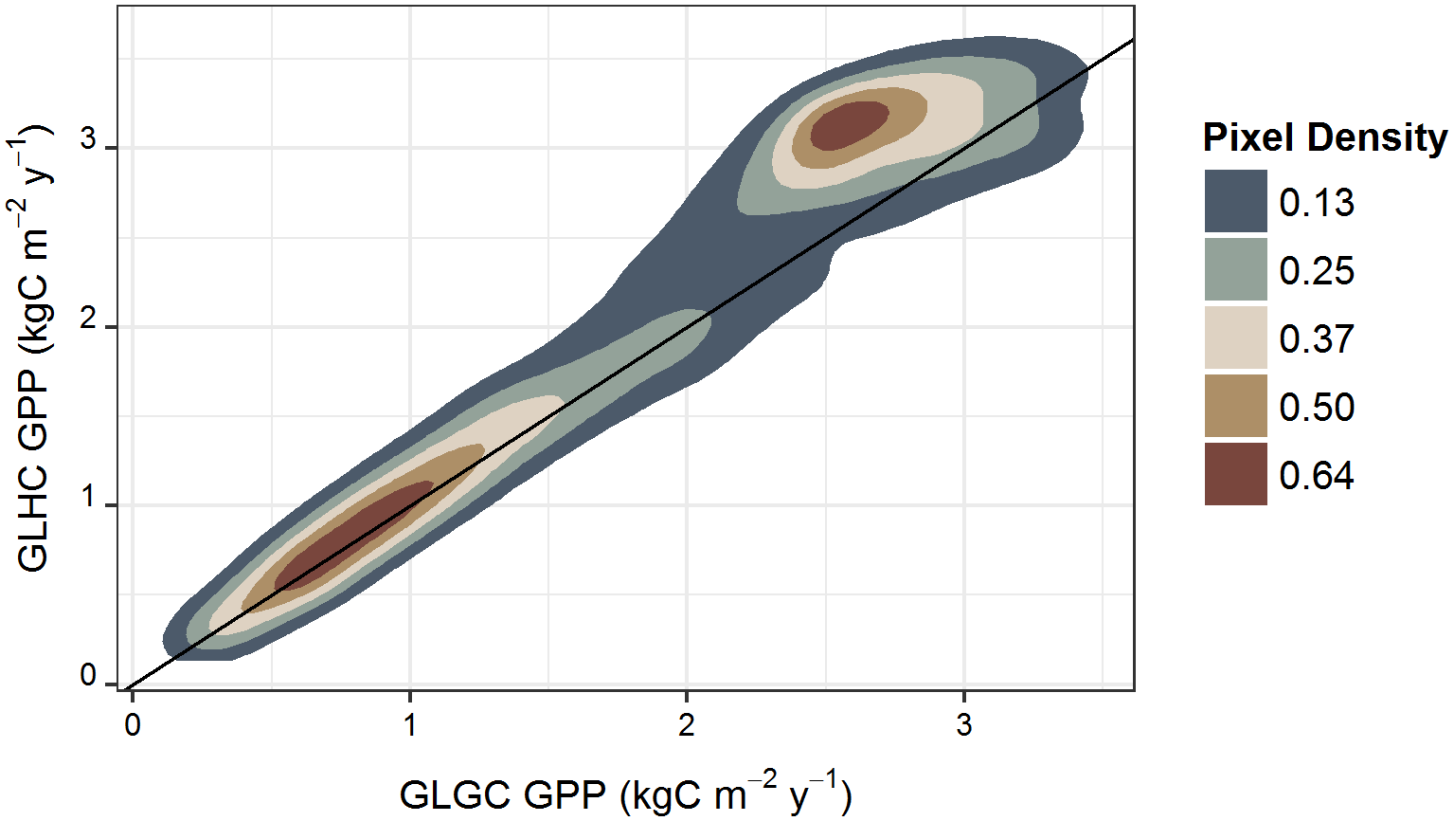
Relationship between GPP estimates using global GMAO climate data (GLGC) and Hawaii-specific climate data (GLHC). This density plot shows the distribution of MOD17 GPP estimates at 500-m resolution using global land cover and climate data products (GLGC) compared to MOD17 GPP estimates produced from the global land cover and high-resolution Hawaii-specific climate data products (GLHC), also at 500-m resolution. Pixel density values are two-dimensional kernel density estimates based on bivariate normal distributions, with higher values corresponding to higher pixel density. The line represents a 1:1 relationship. In high productivity areas, the global climate data products yield higher estimates of GPP than the Hawaii-specific climate products.

There was greater spatial variability in both PAR (Fig 3) and MAT (Table 2) in the high-resolution Hawaii-specific climate products than in the global GMAO products, and statewide mean values of both PAR and annual temperature were higher in the GMAO data than the Hawaii-specific climate data. For both data products, MAT in all vegetated pixels was above the threshold where RUE is subjected to attenuation (S1 Table). The range and standard deviation of VPD values were similar between the Hawaii-specific and GMAO climate data products, but mean VPD was 80% higher in the global GMAO product, and the maximum VPD value was ~45% higher (Table 2). For the Hawaii climate data input product, 75% of the study area had VPD below the minimum value for attenuation of RUE. In areas within the VPD scalar attenuation range (25% of study area), RUE was adjusted downward by 5% or less depending on land cover type. For the GMAO climate data input product, 80% of the study area had VPD values within the scalar attenuation range, where RUE was adjusted downward by 20% or less depending on land cover type. The GLGC model produced higher estimates of GPP in high productivity areas than the GLHC model, but in low productivity areas discrepancies between the two models were smaller (Fig 4).

**Table 2:** Summary statistics from climate data products.

	Min	Max	Range	Mean	Std. Dev.
Hawaii PAR (Wm^-2^)	130	296	167	209	31
GMAO PAR (Wm^-2^)	217	262	45	241	12
Hawaii VPD (kPa)	0.2	0.9	0.7	0.5	0.2
GMAO VPD (kPa)	0.5	1.3	0.8	0.9	0.2
Hawaii MAT (°C)	4	24	20	18	5
GMAO MAT (°C)	18	24	6	22	2

Photosynthetically active radiation (PAR), vapor pressure deficit (VPD) and mean annual air temperature (MAT) for the high-resolution Hawaii and the global GMAO climate input data products.

**Fig 5:**
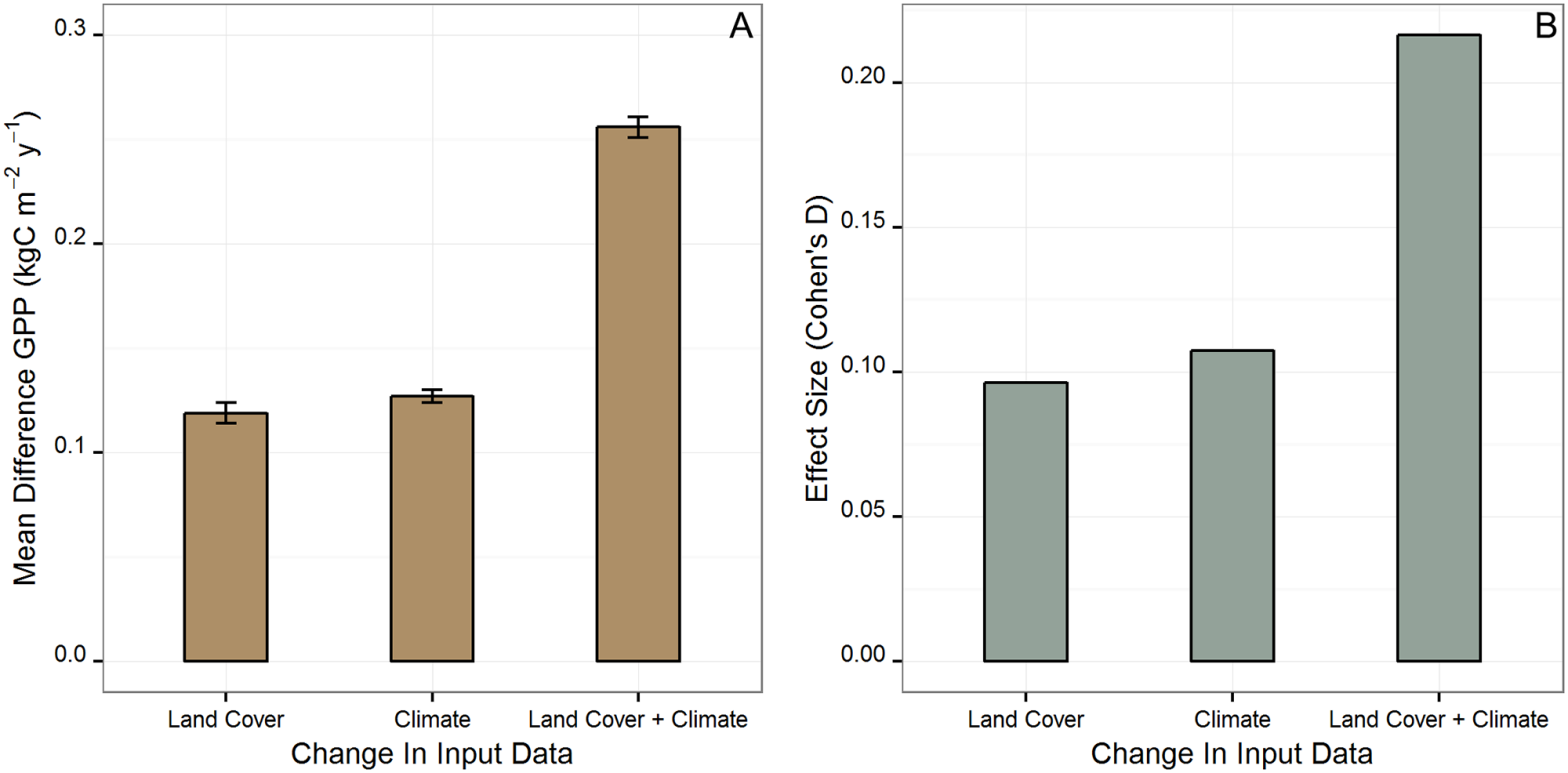
Statewide difference in GPP estimates and effect size of the MOD17 models. (A) The mean per pixel difference in estimated GPP and 95% confidence interval between the global land cover and climate model (GLGC), and the models with Hawaii land cover and global climate (HLGC), global land cover and Hawaii climate (GLHC), and both Hawaii land cover and climate. In each case means were found to be significantly different from zero (p < 0.001, df = 71675, t-values; GLGC/HLGC t = -51.6385, GLGC/GLHC t = -96.5017, and GLGC/HLHC t = -103.0899). (B) The Cohen’s D effect size based the substitution of Hawaii land cover and high-resolution climate data products on MOD17 GPP estimates.

Annual GPP estimated for the state of Hawaii using the combination of global MCD12Q1 land cover and global GMAO climate data products (GLGC) was 28.07 TgC y^-1^ with an average flux density of 2.02 kgC m^-2^ y^-1^ across the archipelago. Replacing MCD12Q1 with the Hawaii land cover product (HLGC) reduced the statewide GPP estimate by 2.14 TgC y^-1^ or 7.6%. The mean difference in GPP between GLGC and HLGC was 0.119 ± 0.005 kgC m^-2^ y^-1^ (Fig 5A) with a Cohen’s D effect size of 0.096 (Fig 5B). Over 95% of the difference in statewide estimated annual GPP based on the two land cover products was driven by pixels classified as sparse or barren in the Hawaii-specific land cover product but designated as having some other vegetation cover in MCD12Q1 (Fig 2A). Using Hawaii-specific climate data products reduced statewide annual GPP by 2.27 TgC y^-1^ (8.1%) compared to estimates derived from the global GMAO climate products. The mean difference in GPP estimated using global GMAO climate products and Hawaii-specific climate products was 0.127 ± 0.003 kgC m^-2^ y^-1^ (Fig 5A), with a Cohen’s D effect size of 0.11 (Fig 5B).

**Fig 6:**
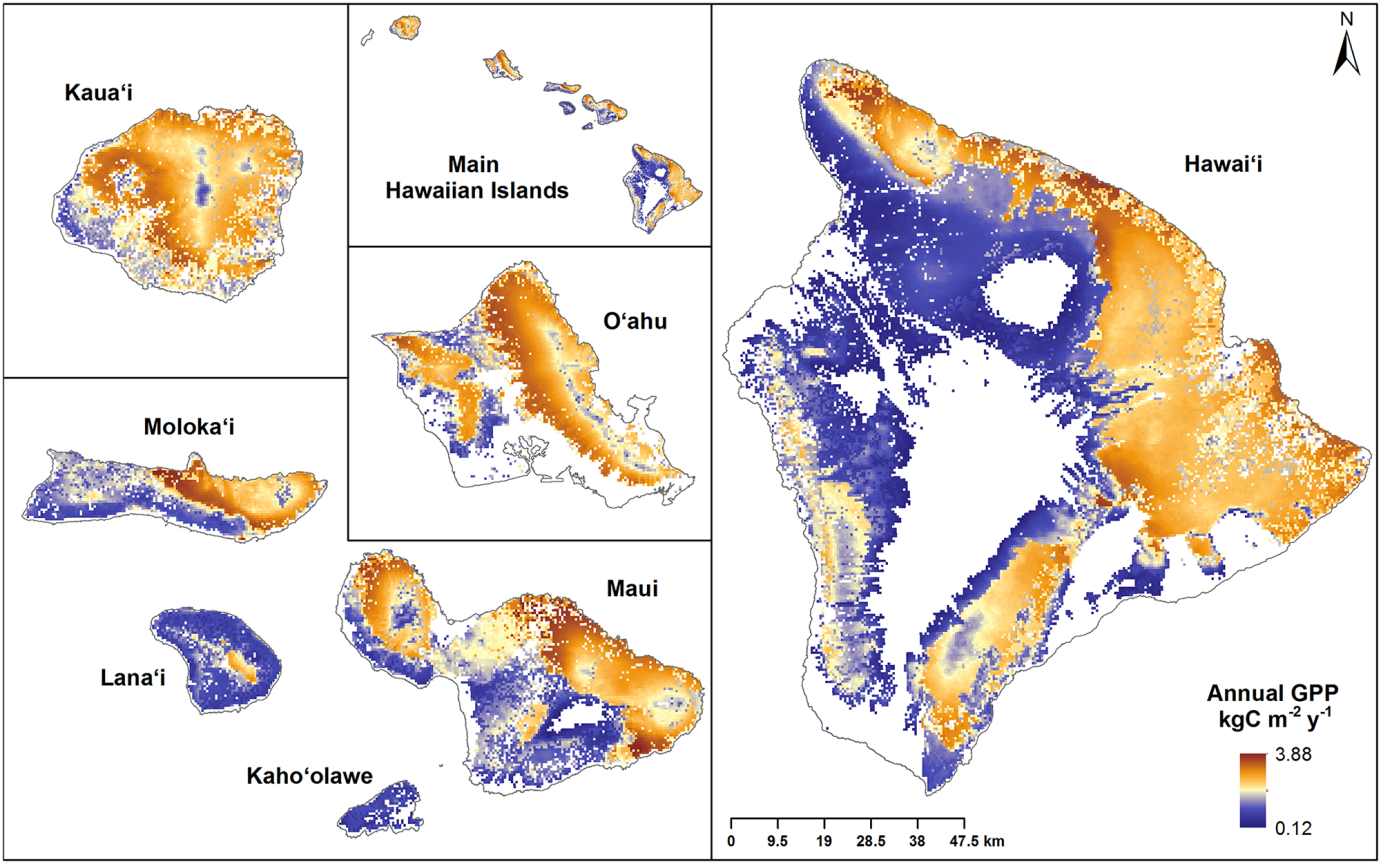
Spatial distribution of GPP estimates for the seven main Hawaiian Islands using Hawaii-specific land cover and climate data products (HLHC GPP Model).

When both global land cover and climate data products were substituted with Hawaii-specific data products (HLHC; Fig 6), we found the statewide MOD17 GPP estimate was 23.47 TgC y^-1^, a reduction of 4.6 TgC y^-1^ (16.4%) compared to the GLGC model. The mean difference in GPP between GLGC and HLHC was 0.256 ± 0.005 kgC m^-2^ y^-1^ with a Cohen’s D effect size of 0.22 (Fig 5A and 5B), roughly double the individual effect sizes of substituting land cover or climate data products individually. These statewide findings were driven by consistent changes in GPP estimates on the four largest islands, which accounted for 92% of the study area. Island-wide GPP for Hawaii Island was affected more by substituting climate products, while GPP estimates for Maui, Oahu and Kauai were influenced more by substitution of the land cover data product (S4 Table).

## Discussion

We evaluated two sources of potential error contributing to uncertainty of MOD17 GPP estimates: land cover and climate data input products. Tropical ecosystems are among the most productive on the planet [13,38], and are also hot spots for land conversion of forest to agriculture or development [39,40]. We focused our analyses on the highly heterogeneous but thoroughly mapped Hawaiian archipelago. Insight into the sources of error contributing to uncertainty of MOD17 GPP estimates for the main Hawaiian Islands should help to clarify future refinement needs for interpretation of MOD17 estimates in other heterogeneous regions, especially those undergoing rapid change in land cover or climate. Resulting improvements may enhance global efforts to reduce greenhouse gases by informing where incentives are best applied to protect and restore forested areas [6].

Our results indicate that the effects of incorporating Hawaii-specific land cover and climate data inputs on MOD17 GPP estimates were additive and independent of each other, with statewide GPP estimates reduced by ~ 8% when either Hawaii-specific land cover or climate data inputs were incorporated, and by ~16% when Hawaii-specific land cover and climate data inputs were used in combination. Although our results suggest that using local land cover and climate data input products may increase the accuracy of MOD17 GPP estimates, we discuss our results only in terms of uncertainty because there are no independent GPP estimates of known accuracy to validate our GPP estimates at individual sites in Hawaii, let alone across the entire study area. The only published ground based eddy covariance data from Hawaii are from two irrigated and fertilized sugar cane fields on Maui during peak growth [41]. These are of limited utility for validation because our GPP estimates are averaged across multiple years and so integrate both fallow and cultivated periods in these pixels. The Hawaiian Islands are not included in the global Max Planck Institute (MPI) GPP dataset, which is based on empirical up-scaling of eddy covariance data from the FLUXNET network [13, 42]. If Hawaii were included, validating our GPP estimates with the MPI dataset would still be problematic because of its coarse 0.5-degree spatial resolution and the lack of any input eddy covariance data from Hawaii. Using solar-induced fluorescence (SIF) data from either the GOME-2 or OCO-2 satellites as a validation dataset is not optimal because SIF-based GPP estimates should be inferred from biome-specific regressions [14], which have not been calculated for any ecosystem in Hawaii. The ideal dataset to validate our GPP estimates and compare the accuracy of our four models would be from a network of eddy covariance towers located across several of the main Hawaiian Islands in all major biomes (forest, grassland, shrubland). Because this network does not yet exist, we are limited to discussing our results only in terms of uncertainty rather than accuracy.

In our comparison of land cover data products, we found only 51.6% agreement between MCD12Q1 and the Hawaii-specific land cover product. Most of this disagreement stems from the scale at which each product was produced. The MCD12Q1 product was produced using a global supervised classification algorithm [17,43], whereas the Hawaii product used a combination of several Hawaii-specific classification processes and data sets, and was developed at a much finer spatial resolution [30]. The lower statewide GPP estimate from the HLGC model was primarily due to the difference in land area designated as unvegetated, which was most pronounced on the larger islands. The Hawaii-specific land cover product had ~1300 km^2^ more area classified as unvegetated than the MCD12Q1 product, manifested as expansions along the edges of unvegetated areas in MCD12Q1 (S1 Fig). If this additional 1300 km^2^ area was truly unvegetated, then we would expect it to have a total GPP value near zero in the GLGC model. However, the same pixels in the additional 1300 km^2^ area classified as unvegetated (GPP = 0) in the HLGC model had GPP values averaging 1.57 ±0.99 kgC m^-2^ y^-1^ in the GLGC model, leading to the higher statewide estimate. We suggest this is because fAPAR and land cover are not completely independent products in the GLGC. In MOD17 collection 6 (GLGC), fAPAR is produced using the global MCD12Q1 land cover product as an input, which explains why fAPAR and GPP values were generated by the GLGC model even in areas classified as unvegetated by the Hawaii land cover data product [44,45].

The reduction in statewide GPP from incorporating high-resolution local climate data was primarily due to differences in the spatial resolution of PAR, which is the dominant climatic factor in the PSN algorithm [46]. Differences in MAT between local and global climate data products did not influence GPP estimates because temperature ranges in vegetated areas were not below the range for scalar attenuation of RUE in either the global or Hawaii-specific datasets. Minimum and optimal temperatures are sensitive parameters for estimating GPP, while maximum temperatures do not have a significant influence on estimates of GPP [47]. For the global climate data, VPD values were within the scalar attenuation range for all the north-western islands and the north-west portions of Hawaii Island, where RUE was reduced by up to 20%, while VPD in the local climate data was below the minimum threshold for scalar attenuation in the majority of the study area (~80%). If this discrepancy in RUE attenuation between the local and global climate data inputs had a large influence on GPP, we would have expected higher statewide GPP estimates using the Hawaii climate product. However, there was an overall reduction in statewide GPP using the Hawaii climate data because PAR is a much more influential variable [47]. Moreover, the higher spatial variability in GPP estimates using Hawaii climate data reflect the spatial distribution of PAR and not VPD (S2 Fig). This was also apparent in the discrepancy between GPP estimates in high productivity areas with high incidence of cloud cover on the wet, windward sides of islands, where the global climate data inputs resulted in higher GPP estimates. Differences in PAR between the Hawaii-specific and global climate data inputs had the largest impact on estimates of GPP for Hawaii Island, the largest of the Hawaiian Islands with the greatest elevation range (sea level to ~4,200 m). The two smallest islands (Lanai, Kahoolawe) have a small elevation ranges, are in the rain shadow of Maui and do not exhibit the same pronounced windward/leeward pattern of PAR, and were thus not as influenced by the differences in PAR input models. Our results suggest the high-resolution Hawaii-specific climate data were better able to capture the spatial variability in PAR in wet, cloud-prone areas of high productivity, ultimately leading to more spatially variable estimates of GPP and lower overall statewide estimates.

We found that the effects of incorporating local high-resolution land cover and climate data on MOD17 GPP estimates for the Hawaiian Islands were roughly equivalent and independent of each other, but we caution that this result may not be generalizable to other heterogeneous tropical landscapes [20,48,49]. For example, the land cover effect on statewide GPP estimates for Hawaii was primarily driven by a difference in the designation of vegetated and unvegetated areas, while misalignment among vegetated land cover classes had little overall impact. However, this should not discount the importance of accurately identifying the spatial distribution and area of different vegetated land cover classes. For analyses specifically focused on land cover conversions between classes with large differences in RUE terms, as between grassland and forest, capturing the area of each land cover class is still critical to estimating GPP [10,19,49]. Similarly, we found that incorporating Hawaii-specific, high-resolution PAR data led to increased spatial variability in GPP estimates and reduced the statewide estimate of total GPP, but that incorporating local MAT and VPD data had little to no impact on GPP estimates. Other areas with higher seasonal and inter-annual variability in temperature and VPD may not yield the same results [19,50,51]. Overall, this study demonstrates a process for quantifying the uncertainty in GPP estimates in heterogeneous tropical landscapes and adds to the body of work that can be used to further refine the MOD17 GPP algorithm. Our findings support previous calls for improved land cover and high-resolution climate input products for the tropics, and identify a need for similar analyses in other regions to enhance our understanding of uncertainty in MODIS based GPP estimates in tropical, heterogeneous landscapes.

## Supporting information

S1 Fig Spatial distribution of the difference in MOD17 GPP estimates between land cover data products. Positive values indicate areas where the Hawaii-specific land cover data product produced higher estimates than the global land cover data product MCD12Q1. Negative values indicate areas where MCD12Q1 produced higher estimates than the Hawaii-specific land cover data product.
(TIF)

S2 Fig Spatial distribution of the difference in MOD17 GPP estimates between climate data products. Positive values indicate areas where the high-resolution Hawaii-specific climate data products produced higher estimates than the global climate data products. Negative values indicate areas where the global climate data products produced higher estimates than the high-resolution Hawaii-specific climate data products.
(TIF)

S1 Table Radiation use efficiency and minimum and maximum attenuation values for land cover classes in Hawaii. (XLSX)

S2 Table Hawaii-specific land cover class designations and corresponding MCD12Q1 designation. (XLSX)

S3 Table Hawaii-specific and MCD12Q1 confusion matrix. (XLSX)

S4 Table Summary statistics for GPP estimates in kgC m^-2^ y^-1^ and total TgC y^-1^ between models by island. (XLSX)
