# Correction: Evaluating the role of land cover and climate uncertainties in computing gross primary production in Hawaiian Island ecosystems

**DOI:** 10.1371/journal.pone.0192041

**Published:** 2018-01-25

**Authors:** Heather L. Kimball, Paul C. Selmants, Alvaro Moreno, Steve W. Running, Christian P. Giardina

The labels of the x- and y-axis in Fig 4 are reversed. Please see the correct Fig 4 here.

**Fig 4:**
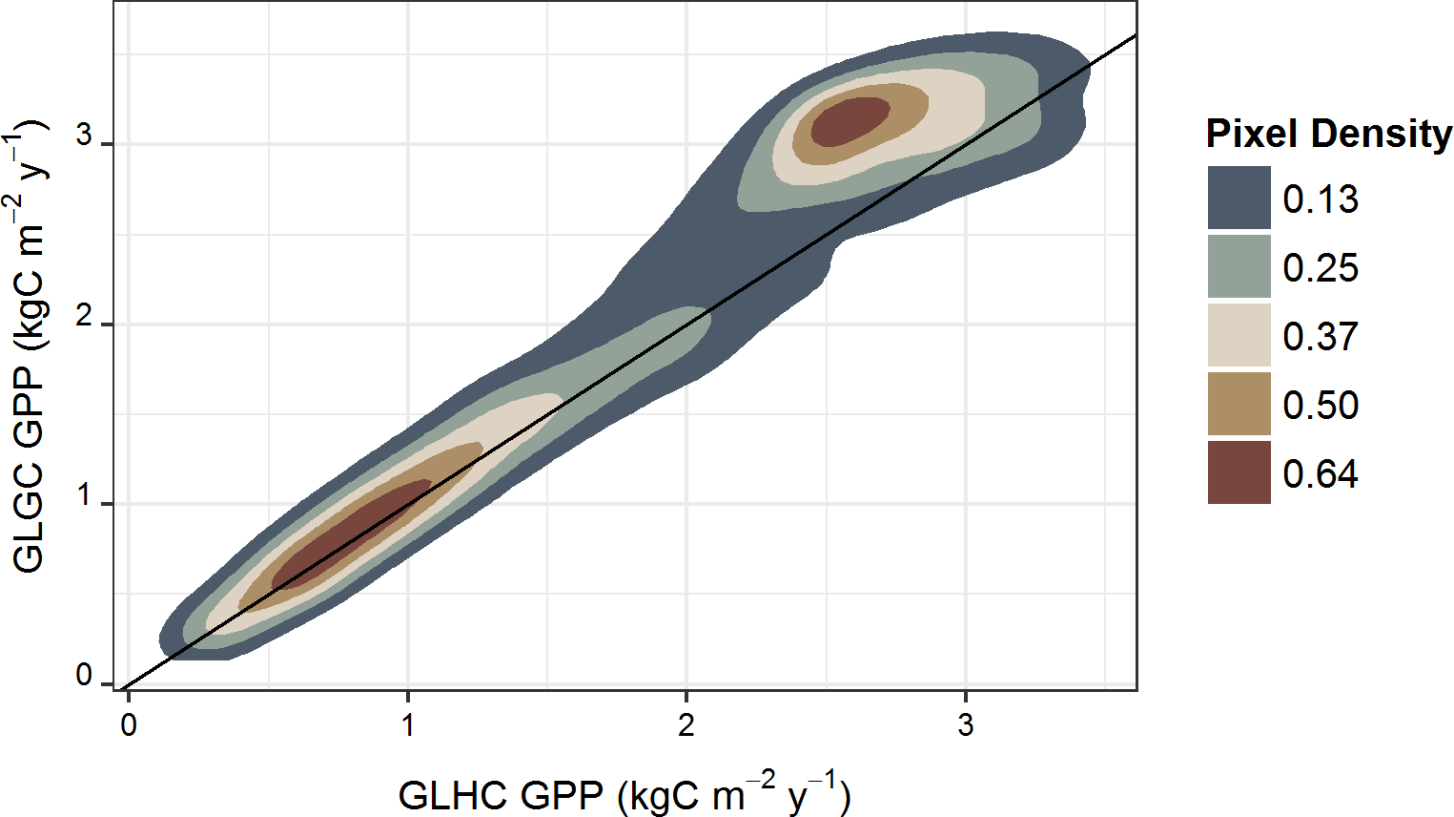
Relationship between GPP estimates using global GMAO climate data (GLGC) and Hawaii-specific climate data (GLHC). This density plot shows the distribution of MOD17 GPP estimates at 500-m resolution using global land cover and climate data products (GLGC) compared to MOD17 GPP estimates produced from the global land cover and high-resolution Hawaii-specific climate data products (GLHC), also at 500-m resolution. Pixel density values are two-dimensional kernel density estimates based on bivariate normal distributions, with higher values corresponding to higher pixel density. The line represents a 1:1 relationship. In high productivity areas, the global climate data products yield higher estimates of GPP than the Hawaii-specific climate products.
