# Supplementary figures and images for: Evaluating the role of land cover and climate uncertainties in computing gross primary production in Hawaiian Island ecosystems
Source: PLoS One. 2017 Sep 8;12(9):e0184466. doi: 10.1371/journal.pone.0184466 (PMC5590934; doi:10.1371/journal.pone.0184466)

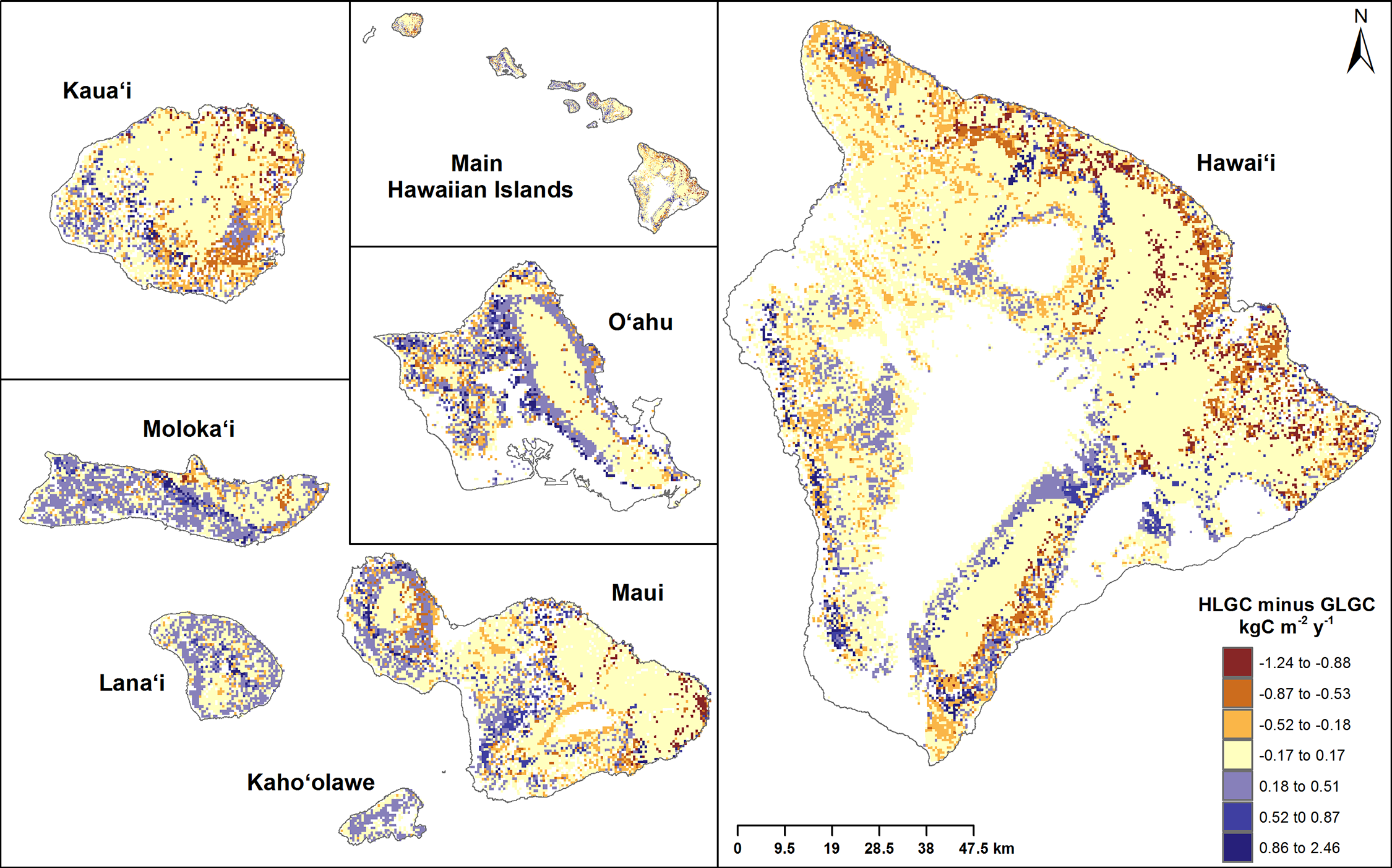

Supplement: S1 Fig — Positive values indicate areas where the Hawaii-specific land cover data product produced higher estimates than the global land cover data product MCD12Q1. Negative values indicate areas where MCD12Q1 produced higher estimates than the Hawaii-specific land cover data product. (TIF) [file pone.0184466.s001.tif]

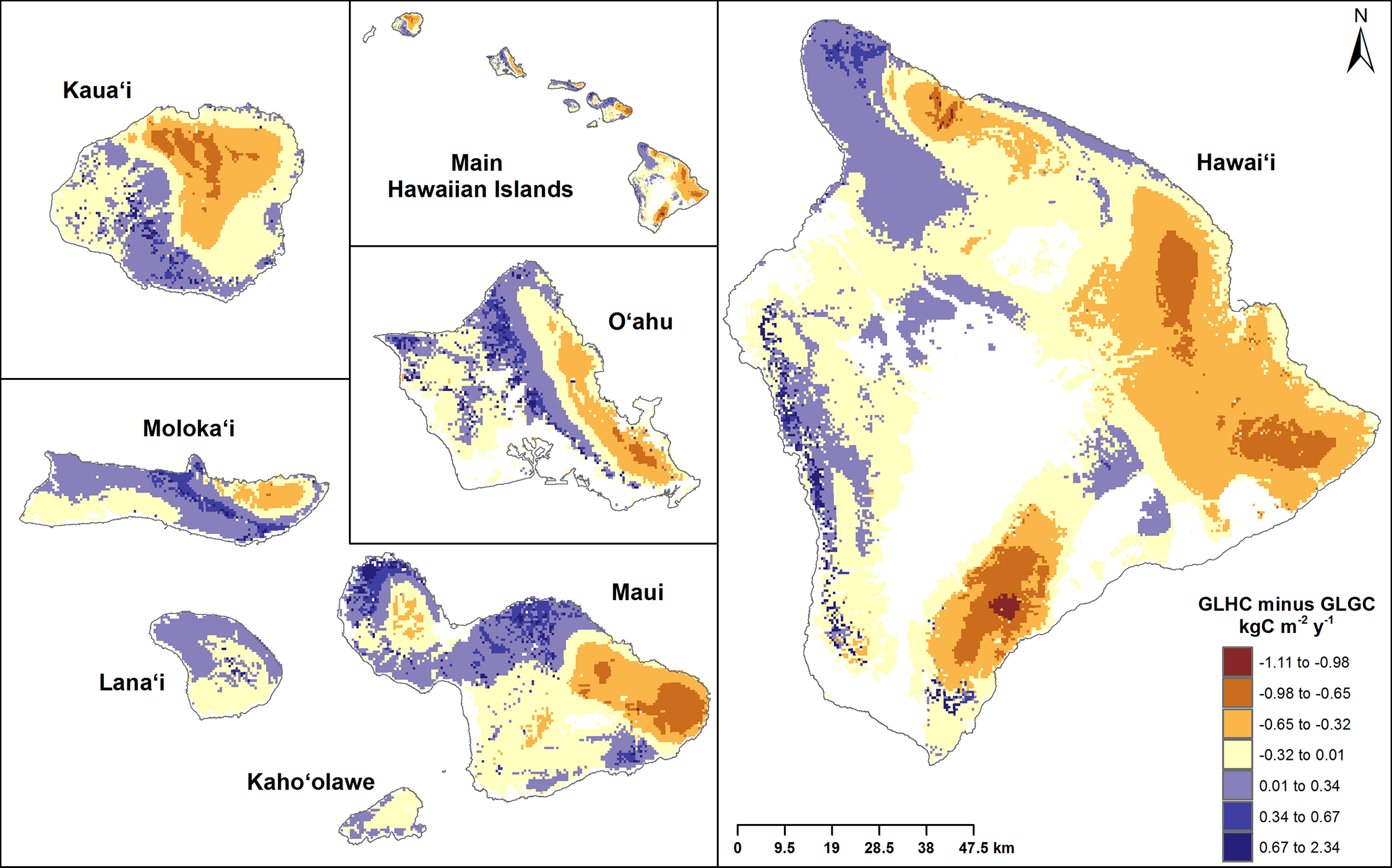

Supplement: S2 Fig — Positive values indicate areas where the high-resolution Hawaii-specific climate data products produced higher estimates than the global climate data products. Negative values indicate areas where the global climate data products produced higher estimates than the high-resolution Hawaii-specific climate data products. (TIF) [file pone.0184466.s002.tif]
